# Well-being and relative deprivation in a digital era

**DOI:** 10.1016/j.heliyon.2022.e11233

**Published:** 2022-10-24

**Authors:** Zahra Hatami, Sue Yi, Margeret Hall

**Affiliations:** a College of Business, University of Louisville, United States of America; b School of Interdisciplinary Informatics, The University of Nebraska at Omaha, United States of America; c Strategy and Innovation Department, Wirtschaftsuniversität Wien, Austria

**Keywords:** Davies J-curve, Well-being, Relative deprivation, Human development index, Network analysis

## Abstract

Social unrest is a feature of the early 21st century, yet relatively little research binds theoretical aspects with empirical validation of the drivers of protests and revolutions. This study aims to empirically validate the Davies J-Curve considering the digital era, with economic, social, and political factors. Using big data techniques, network analysis, and theoretical analysis, we analyzed countries' similarities by analyzing Human Development Index (HDI) and Worldwide Governance Indicator (WGI) as proxies of social well-being. Results established the existence of a J-Curve during social crises in countries prior to an occurrence of large-scale social unrest. In addition, our results suggest that HDI was not a sufficient indicator regarding countries' experienced well-being, likely because it is missing the highly granular aspects of daily life. We further recommended that other indicators from political and psychological areas should be considered and treated in the data preparation phase for future society-wide well-being research for a more realistic baseline.

## 1 Introduction

Non-violent and violent protests are increasingly common in recent years across continents and national development levels. Argued in the digital public square and equipped with real-time streaming capabilities and marginal reproduction costs, individuals are airing grievances on declining living standards, perceived radical demographic shifts, and universal displeasure with the in-place governing institution. In 2019 alone, countries as diverse as Bolivia,^1^ Chile,^2^ France,^3^ Iran,^4^ Hong Kong,^5^ and Iraq^6^ witnessed large-scale protests with critical disruptions to public life. Economic reasons such as inflation are particularly common factors in which a relatively small blow to living standards (e.g., an increase in metro ticket prices in Chile or an increase in gas price in Iran) targeted people's ability to survive in highly unequal societies. However personal economics alone is not the only factor in taking displeasure with the status quo to the streets and the digital public square (Afzali et al., 2019). Since the advent of public health measures linked to the global COVID-19 pandemic, public protests have only increased in size, frequency, and location culminating in violent events like the mass storming of the US Capitol by protesters demonstrating against the election of US President Joseph Robinette Biden in 2021 and the grudge-based 2022 assassination of former Prime Minister Shinzo Abe of Japan.

Technology has accelerated the process of protests since organizing and maintaining protests with increasing usage of the internet is easier than in the past (Poell, 2019; Tufekci and Freelon, 2013). For example, the widespread availability of smartphones gives protesters not only the ability to interact locally for distributing information and for encouragement (Boecking et al., 2015; Skoric, 2012), but technology also increases their ability to compare their lives and livelihoods, and compare across nascent movements globally (Bimber, 1998; Treré and Mattoni, 2016; Bennett and Activism, 2003). While it is clear that international economic and demographic confluence and wide-spread digitalization are transforming the political economy, the extent to which this is happening is currently unknown. In his foundational article, Towards a Theory of Revolution, James Davies argued that economic factors are the agent of revolution (Davies, 1962). His main assumption is that when a long span of economic and social growth results in rapid reversal, a revolution is more likely to occur. Long-term growth raises expectations, and the short-term downturn creates an intolerable gap between expectations and incomes and, as a result, social unrest. The theory rests heavily on the idea of relative deprivation, or feeling deprived compared to expectations or observations of others. This observation is a powerful theory about why those with objectively low quality of life (i.e., low life expectancy, low educational attainment, high inflation, low income) may not revolt as opposed to those with overall higher well-being (i.e., high trust in government, widespread access to the internet, developed economies), may take part in protests. While the J-Curve has generated considerable academic discussion since its introduction in 1962 (Gurr, 1970; Geschwender and Geschwender, 1973; Bremmer, 2006; Orbell and Shay, 2011; Snyder and Tilly, 1974) it has yet to be consistently empirically demonstrated (e.g. (Bernstein and Crosby, 1980; Knutsen, 2014; Miller et al., 1977; Smith and Pettigrew, 2014). Moreover, most literature analyzed and reported their findings before the Internet permeated society. Given the Internet's role in deconstructing and reconstructing industries and economies, coupled with its potential use as a comparative platform we propose re-opening the discussion on the applicability of this theory to predict large-scale protests and revolutions in the digital era.

This research utilizes network analysis to understand if countries that are going through revolutions are share indicators at the macroeconomic level, whether they considered are stable or unstable, and whether they follow the J-Curve or not. Ten indicators are the basis of the analysis (see Methodology). The United Nations Human Development Index (HDI) was the main indicator regarding creating the networks and comparing countries' well-being along with the Worldwide Governance Indicator (WGI) from the World Bank. Inflation, the life ladder/Happiness Index, national-level internet penetration statistics, and six governance indicators (voice and accountability, political stability, government effectiveness, regulatory quality, rule of law, and control of corruption) are used for performing cluster analysis. We applied network analysis to the Davies J-Curve to evaluate the following research questions:

RQ1-Considering subjective and objective measures of human development and well-being, which indicators are most suitable for modeling quality of life?

RQ2-What are the similarities between countries regarding quality of life indicators in a cluster?

RQ3-Which recently unstable countries follow the J-Curve?

RQ4-Which indicators drive stability or instability over time?

We theoretically ground the analysis in the Related Work section, followed by a description of the data and the analytical approach. We continue with the results of the network analyses and an in-depth look at the J-Curve across data types. The article contextualizes and grounds the results into the on-going discussion around the applicability of the J-Curve to empirically model large-scale protests, and concludes with a discussion of limitations and future work.

## 2 Theoretical background

### 2.1 Well-being

Well-being is a fuzzily defined construct as it is subjective, multi-dimensional, and dynamic in measurement. White and Pettit (White and Pettit, 2004) stress the normative aspects of the concept. Well-being and its assessment are inevitably based on values and judgment made up of states like material endowments, psychological attributes, and subjective assessments of the personal and environmental (2004). Well-being can be evaluated as subjective well-being, psychological well-being, access (i.e., internet, utilities), or via economic utility functions (Anand and Sen, 1994; Di Tella et al., 2001; Diener and Suh, 1997; Helliwell and Huang, 2008; Karlsson et al., 2004; Romer and Romer, 1998; Ryan and Deci, 2001; Stiglitz et al., 2009; Waterman, 2007; Zamagni, 2014). Each perspective has varying strengths; as complimentary assessments a fitting proxy of individual and institutional well-being can be developed (Gasper, 2005; Huppert and So, 2013; Samman, 2007; Stiglitz et al., 2009; Lindner et al., 2015).

Flourishing people and institutions share some characteristics including higher productivity, more effective learning, stable social ties including more pro-social behaviors and better relationships, and stronger health and life expectancy's (Diener and Chan, 2011; Frey and Stutzer, 2012; Huppert and So, 2013). These attributes create multiplier benefits across society. People and societies reporting higher well-being also have lower expenditures on programs to curb social disintegration, lower healthcare costs, and overall higher quality of living (Gasper, 2005; NEF, 2009; Oishi et al., 2007).

Assessing well-being as economic utility equates tangible metrics including personal income, wealth, safety and social security to well-being. Well-being economists assume that with a minimum or higher level of such measures that individuals can achieve well-being as a function of personal fulfillment. As they are tangible and relatively easy to measure, and widely used in support of political decision making, economic perspectives of well-being are popular (Ahn et al., 2011; Frey and Stutzer, 2007; Stutzer and Frey, 2012). However, economic well-being measures are not granular enough to provide insights about personal well-being levels. They are rather used to assess and compare well-being on a more general, national basis. Using income and material wealth as the sole definition of economic utility has long been recognized as a flawed approach (Bentham, 1970; Smith, 2010). Even with cautions from foundational thinkers like Adam Smith and Jeremy Bentham, multiple studies support a correlation between economic well-being and personal well-being on a macroeconomic scale (Stevenson and Wolfers, 2008; Diener and Seligman, 2004). Scholars making this connection argue for the importance of economic measures to assure well-being particularly when considering basic need fulfillment, though they relativize the importance of this link in highly developed countries. This is what has come to be defined as the ‘Easterlin Paradox.’

The Easterlin Paradox describes a saturation point in the relationship between income and well-being on a national basis (Easterlin, 1974). In developing countries, well-being and income have a positive relationship in that happiness increases with income. Upon reaching a saturation point of annual income (estimated as $10,000 at the time), well-being and income endures a negative relationship. Easterlin explains the paradox as the decreasing importance of additional units of income after basic needs are satisfied (Stevenson and Wolfers, 2008). Several studies have confirmed this finding when comparing individual countries and in time series analyses of averaged national economic data (Blanchflower and Oswald, 2008; Kahneman et al., 2006; Easterlin, 1995) but is not uniformly accepted. Competing economic research reports other findings (Gasper, 2005; Preziosi, 2013). The competing studies tend to be smaller, and are less widely accepted due to methodological reasons (Easterlin et al., 2010). The link between personal and national economics and well-being is also reflected in the United Nation's Human Development Index (HDI) in their assessment of human well-being (Stiglitz et al., 2009). Very much reflective of the cliché that rising tides lift all boats, the currently most broadly accepted measure considers not personal well-being but rather the social and institutional aspects which are conducive to high quality of life.

### 2.2 Governance and well-being

Governance has been defined as “the traditions and institutions by which authority in a country is exercised. This includes (a) the process by which governments are selected, monitored and replaced; (b) the capacity of the government to effectively formulate and implement sound policies; and (c) the respect of citizens and the state for the institutions that govern economic and social interactions among them.” (Kaufmann et al., 2010) (page 4). The quality of institution, or governance has been linked to well-being. The relationship between governance and well-being have been investigated at the national level (Helliwell et al., 2018), through human development (Nandha and Smyth, 2013), by comparing economic differences across countries (Woo, 2018), and by investigating the efficiency of governmental policies (Debnath and Shankar, 2014). Improvements in the quality of governance has been found to lead to greater average levels of citizen well-being (Helliwell and Huang, 2008; Ott, 2010, Ott, 2011). This linkage has been shown to be correlated economically in that, when government expenditure to GDP increase, life satisfaction decreases (Bjørnskov et al., 2007).

Further, Frey and Stutzer famously empirically showed the impact of good governance directly or indirectly when comparing the life satisfaction of Swizz residents and non-Swiss residents living in the different states of Switzerland (Frey and Stutzer, 2005). Their results demonstrate that people report being better off when living in the context of good government. One reason is the direct effect of “procedure utility”, the notion that people care about outcomes in addition to preserving the right to participate in political decision-making processes (Frey, 2008; Ott, 2010). When people feel that they can participate, they are happier overall.

### 2.3 J-curve and well-being

**Figure 1:**
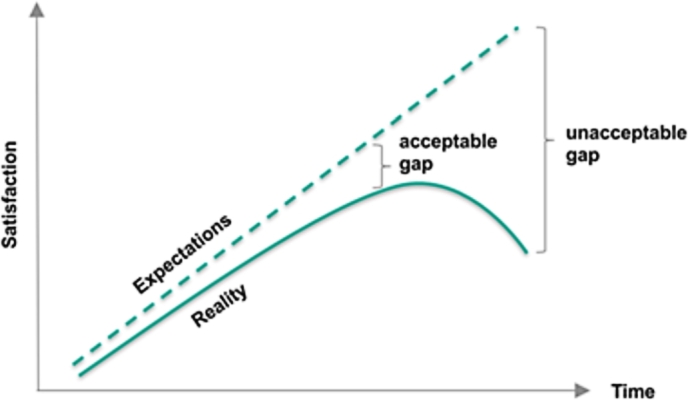
The Davies J-Curve.

The J-Curve serves as a useful model for the utilization of well-being as a policy indicator not for positive impacts, but for the negative impacts of social unrest. James Davies suggested in his foundational work that drops in subjective expectations of quality of life as compared to actual progress fuels relative deprivation (Davies, 1962). Relative deprivation is also subjective: it is a type of deprivation experienced when only in comparison to others who are more(less) fortunate (1962). In his model, significant differences between actual and expected advancement reveals the overall well-being and vigor of the society or institution. Otherwise stated, social unrest is a subjective response to a sudden reversal in fortunes after a long period of growth (see Fig. 1).

If the model can be expected to be prescriptive or predictive, relative deprivation must be evaluated by charting the change in metrics of actual well-being levels against expected well-being assessments. When comparing expected and actual well-being levels, absences of statistically significant differences implies no serious discrepancy, but instead, suggests a lack of social unrest. Significant differences imply the opposite. This key research concept is supported by the model: if relative deprivation is not occurring, social turmoil in turn does not occur regardless of the actual state of well-being and quality of life.

### 2.4 Relative deprivation

In 1949, Stouffer and colleagues introduced the concept of relative deprivation (RD) (Stouffer et al., 1949). The central concept of RD is that when individuals perceive themselves as worse off than others, the individual will experience negative emotions (Runciman and Runciman, 1966), from frustration to alienation to anger and dissatisfaction (Smith et al., 2012). Ted Gurr positioned RD in terms of the “actors' perception of discrepancy between their value expectations and their value capabilities. Value expectations are the goods and conditions of life to which people believe they are rightfully entitled. Value capabilities are the goods and conditions of life they think they are capable of getting and keeping” (Gurr, 1970) (page 13). Early research in RD was largely theoretical but during the following decades, RD has evolved to explain a variety of social phenomena (Crosby, 1976; Mark and Folger, 1984; Walker and Smith, 2002), in particular, social comparison and social identity theory (e.g. (Crosby, 1982; Folger, 1987; Walker and Pettigrew, 1984). Deprivation may be an individual or collective experience (Runciman and Runciman, 1966). One may believe that she or he is deprived, which is associated with interpersonal attitudes and behaviors, such as well-being and satisfaction. On the other hand, the individual may belong in a group that is deprived, which is associated with intergroup attitudes and behaviors such as political participation or protest (Walker and Pettigrew, 1984; Pettigrew et al., 2008; Pettigrew and Meertens, 1995; Osborne and Sibley, 2013). Ultimately, RD is a classic social psychological concept that underlies the potential for revolutions to occur.

### 2.5 Social unrest and revolution

That individuals may use digital media in support of social unrest is well-established (Tufekci and Freelon, 2013; Boecking et al., 2015). Unknown is the degree to which it is creating relative deprivation, which can lead to social unrest. The IMF defines social unrest as protests, riots, and other forms of civil disorder and conflict which may be sporadic and inconsistent or sustained.^7^ We use this term to encompass varying forms of instability in a country leading successful or unsuccessful revolutions. There are several definitions of “revolution” offered by social science theorists such as (Davies, 1962; Gurr, 1970; Skocpol, 1979). These definitions often imply a common characteristic to revolutions in that the social upheaval must be successful. Tilly stated that revolutions bring about a revolutionary outcome (subordinate regime successfully displace the elite regime) (Tilly, 1977). Another implied characteristic of revolutions is the use of violence. Samuel P. Huntington's widely applied definition of revolutions are “a rapid fundamental and violent domestic change in the dominant values and myths of a society, in its political institutions, social, structure, leadership, government activity, and policies.” (Huntington, 1968) (page 264). For our study, we will follow Kimmel's definition of revolutions as “attempts by subordinate groups to transform the social foundations of political power” (Kimmel and Kimmel, 1990) (page 6). This definition is inclusive of successful and unsuccessful revolutions. Moreover, it allows inclusion of non-violent protests but still aim at large-scale change by a minority against a perceived majority group.

## 3 Methodology

To support our assessment of the existence of the J-Curve in the digital era, we utilize correlation network and cluster analyses. The correlation network approach is appropriate for problems that involve time series where correlation plays an important role like finance (Tse et al., 2010; Hatami et al., 2022c, Hatami et al., 2022b, Hatami et al., 2022a), social computing (Hatami et al., 2021), bioinformatics (Dempsey and Ali, 2014), and others. A network is a collection of vertices and edges, N = (V, E) (Gao et al., 2013); Vertices or nodes are entities and the relationship between entities is represented by edges if, to a certain degree there is a similarity between entities' specific parameters. For example, in the finance domain, companies (entities) could be represented by nodes, and similarities between companies' prices or returns are represented by edges.

Correlation Network Models (CNM) are useful model to assess macro-trends across countries as the highly correlated nodes or nodes with dense connections would give us information about the countries with the same kind of behavior or characteristics. In this research, CNM are built by assuming that each country is a node (a vertex) in the graph model and two countries are connected by an undirected edge if and only if their correlation coefficient is 90% or more. As the data at hand are normally distributed, Pearson correlation network was applied in the network (Hatami et al., 2021).

In the next step we turn to cluster analysis. Cluster analysis is a data analysis shortcut tool that aims to arrange different objects into groups whose degree of correlation between two objects is maximal (Mousavi and Nagy, 2021). We used cluster analysis as a statistical method for grouping countries, according to their similarity in their Human Development Index (HDI). Through cluster analysis and cluster enrichment, the countries are divided into distinct, homogeneous clusters.^8^ This method is used to segment countries based on their similarities.

We created and analyzed three different networks based on the HDI for three different sets of countries. The first network created for all countries around the world where full indicator data was available (n=165). The second network created for the 40 countries in the Organization for Economic Co-operation and Development (OECD). The OECD is widely considered to be representative of stability, though several member countries experienced wide-spread protests in the close of the 2010's. The final network investigated 21 countries that experienced large-scale public protests in 21st century We particularly investigate what, if any, aspects correspond to the J-Curve formation.

### 3.1 Data acquisition and filtering

This research considers fundamental factors in well-being. Well-being can be related to outcomes in economic, social, education, health, and income equality (cf. Related Work). We additionally included access to the internet as other relevant factor that impact people's satisfaction and interactions. In line with (Davies, 1962), if a society has a socioeconomic achievement for a relatively long time and then it goes into rapid decline, then the society should experience public turmoil. However, societies differ in terms of morals, cultural characteristics, historical background, and so on. Therefore, different paths may be found during the revolutions. Moreover, with globalization came widespread technological advances and accessibility; technology being hailed as one of the enabling forces of the waves of social revolution throughout the countries in different regions (Poell, 2019). Data were collected from the World Bank, the United Nations Development Programme (UNDP), Global Change Data Lab^9^ and the OECD. We plot the data starting from the year 2010 through 2017 because for analyzing data and tracking factors involved in the network, we need to have all data points available congruently. Due to the unavailability of data for some countries, we are restricted to the years 2010 to 2017.

#### 3.1.1 Socio-economic indicators

**Figure 2:**
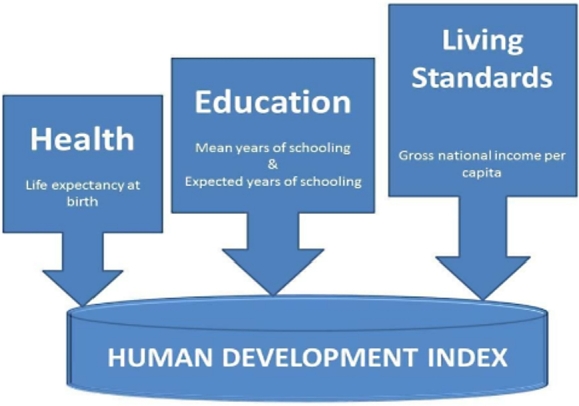
Components of the Human Development Index as proposed by the United Nations.

**HDI** is a composite statistical index measuring life expectancy, education, and per capita income. These three indices are used to rank countries into four tiers of human development covering low to very high development. HDI is calculated by the UNDP as “goalposts” between 0 and 1, in three dimensions: Health, Education, and Living Standards, as indicated in Fig. 2.

Each area of HDI aims to capture the main facets of human development: longevity, knowledge and access to resources. This index is inherent in the measurement of the country's achievements in terms of health and longevity, education, and actual income of its citizens (Mankiw et al., 1992). HDI is not the sole representative index of development, nor is it universal. It does, however, represent an attempt to normalize standards worldwide and has wide data availability allowing for a quantifiable method to look at the satisfaction of the expected need of the population over time. These three dimensions of human development have been the only indicators since its onset. Measuring expectations and actual well-being are difficult without having completed a baseline data assessment and receiving real-time data. To combat this problem and for the purposes of only this initial assessment, we defined expected well-being in terms of change in HDI scores over time. When seen from this paradigm, changes in the overall well-being of the state are driven from the aggregate number of citizens in the state and their access to civil services, reflecting the view of economist and Nobel laureate Amartya Sen that human development is the impact and richness of the human life, rather than merely economic advances.

The HDI sub-indicators are as follows:

**Life expectancy at birth**^10^ refers to the average number of years a newborn is expected to live if mortality patterns at the time of its birth remain constant in the future.

**Mean years of schooling**^11^: Average number of years of education received by people ages 25 and older, converted from educational attainment levels using official durations of each level.

**Expected years of schooling**^12^ are the number of years during which a 2-year-old child can expect to spend in schooling, based on the school enrollment rates at a given date. These expected years are calculated as the sum of enrollment rates observed at the different ages from 2 to 29 years old.

**Gross National Income(GNI) per capita**^13^ is converted to international dollars using purchasing power parity rates (PPP). An international dollar has the same purchasing power over GNI as a U.S. dollar has in the United States. Additional indicators are included to measure areas of well-being that HDI currently does not measure, but are included in our analyses are:

**Inflation**^14^ is measured by the consumer price index reflects the annual percentage change in the cost to the average consumer of acquiring a basket of goods and services that may be adjusted or changed at specified intervals, such as yearly.

**Internet users**^15^ are individuals who have used the Internet (from any location) in the last three months. The Internet can be used via a computer, mobile phone, personal digital assistant, games machine, digital TV, etc. This is assessed yearly.

**Life ladder**^16^ (“Happiness Index”) is the subjective measurement of well-being with reasonable accuracy gained from longitudinal survey data gathered nationally. The measurement score numbers from 0 to 10 at the top. The top of the ladder represents the best possible life, and the bottom of the ladder represents the worst possible life.

#### 3.1.2 Governance indicators

The Worldwide Governance Indicators (WGI), calculated since 1996 by the World Bank, assesses the state of governance in a country (Kaufmann et al., 2010). Using 31 different sources, the WGI encompasses governance on over 200 countries source using six broad dimensions, which are explained below:

**Voice and Accountability** (VA) captures perceptions of the extent to which the citizens are able to participate in selecting their government, as well as freedom of expression, freedom of association, and free media (Pan et al., 2020) (PAGE 9).

**Political Stability and Absence of Violence/Terrorism**(PV) measures perceptions of the likelihood that the government will be destabilized or overthrown by unconstitutional or violent means, including politically motivated violence and terrorism (Kokol et al., 2019). (PAGE 591)

**Government Effectiveness** (GE) measures the quality of public services, civil service, policy formulation, policy implementation and credibility of the government's commitment to raise these qualities or keeping them high (Mariadas et al., 2021). (PAGE 3)

**Regulatory Quality** (RQ) captures perceptions of the ability of the government to formulate and implement sound policies and regulations that permit and promote private sector development (Tatar et al., 2021) (PAGE 5).

**Rule of Law** (RL) measures countries' rule of law performance across eight factors: Government Powers, Absence of Corruption, Open Government, Fundamental Rights, Order and Security, Regulatory Enforcement, Civil Justice, and Criminal Justice (Adaman et al., 2018)(PAGE 169).

**Control of Corruption**^17^ (CC) measures the extent to which public power is exercised for private gain, including both petty and grand forms of corruption, as well as “capture” of the state by elites and private interests.

#### 3.1.3 Correlation network and community detection

To study the features of the correlation network for countries, we first establish the correlation network by inputting the change in HDI over time for three different sets of countries is recorded as an input matrix. In the matrix, each row is a country that contains 8 years of HDI from 2010-2017. In other words, there are 8 data observations for each country in the matrix and n=165;40;21 variables respectively. The reason behind the creation of the three networks was to first determine if HDI is a suitable benchmark for comparing countries regarding well-being and the creation of the J-Curve. We input governance, happiness index, inflation, internet usage, and HDI indicators separately.

To maintain high correlation between countries, we used the 90% threshold as a suitable correlation coefficient. It means each country, represented as a node (vertex) in the graph model, will be connected to another country with an undirected edge if and only if their correlation coefficient is 90% and the significance value of the relationship is at 0.05 or higher (Hatami et al., 2021). This creates a Correlation-Network with countries as nodes (highly correlated countries) connected by edges. There were 165, 40, and 21 countries in the proposed networks. GLay community clustering is applied to the network with all default parameters as established in Cytoscape (Shannon et al., 2003) to produce clusters. GLay clustering was used since it identified as an efficient clustering algorithm between people how work on complex network analysis such as bio-science. As the communities were formed with high correlations among the nodes, we could infer that the overall behavior of the nodes within each community is highly similar. Since GLay communities are the sub-networks, all parameters were added to each community for further analysis for the purpose of doing cluster enrichment for that specific community (Hatami et al., 2021). In the final step of the analysis, we compared countries for HDI's dimensions, political measurements, inflation, happiness, and internet usage and investigate if the countries follow Davies' J-Curve, and if so, on which metrics they do so.

## 4 Experimental results

**Figure 3:**
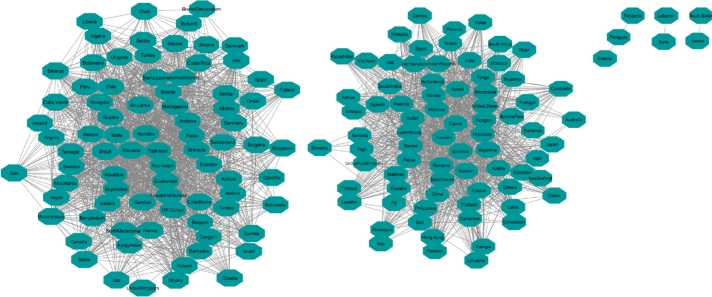
HDI Network Nodes represent the countries and the relationship between them are represented by edges.

**HDI Network**:

The data collected from UNDP contains data on 177 countries around the world; of these, 165 have complete data. By applying the 90% correlation coefficient for finding the similarities between countries' HDI during 2010-2017, 165 countries are mapped in the network. Two large and three small clusters are the result of the GLay clustering algorithm (Fig. 3). The network and clusters extracted from the network show that based on HDI, stable and unstable countries are grouped together in the same cluster. It implies that based on this indicator, stable and unstable countries should have a similar level of well-being.

There were 82 and 76 countries in the two top clusters, respectively. To eliminate sampling bias, each cluster has 10 countries randomly selected to include as a sub-analysis comparing attributes of stable and unstable countries. To identify the differences between stable and unstable countries, the average rate of all indicators, inflation, internet users, happiness, and six governance indicators (VA, PV, GE, RQ, RL and CC) were compared across those countries. Table 1 displays the sample countries in Cluster One with an average of their indicators during 2010-2017. This table confirms that in Cluster One, based on HDI, stable and instable countries are grouped together. In this table, Ireland, Canada, Denmark, Switzerland, Sweden, which are regarded as highly stable countries(green color) are in the same community network with Iran, Congo (DR Congo), Chad, Tunisia, and Ukraine which are unstable countries(orange color). Even as these countries come to one cluster because they have the most similar HDI values, they are totally different in their behavior from political, social satisfactory and economic perspectives (Table 1). Applying cluster enrichment (which means importing average of all indicators to corresponding countries) shows that stable countries have higher levels of VA, PV, GE, RQ, RL, CC, Internet Usage, Happiness and lower inflation compared to unstable countries. Following the indicators with corresponding countries indicate that while HDI may measure general progress, it is not a suitable indicator for differentiating stable and unstable countries.

**Table 1:**
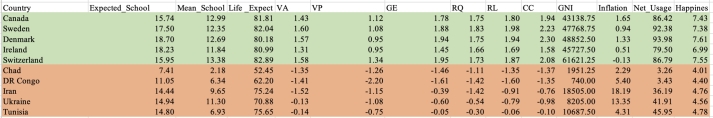
Sample of ten countries from Cluster One.

**Figure 4:**
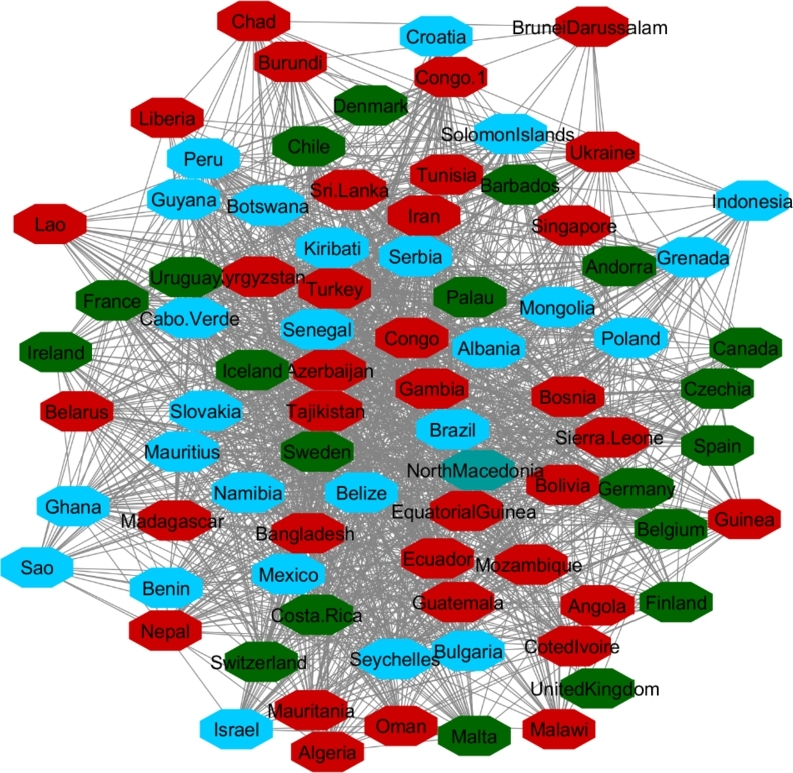
Happiness levels for Cluster 1 where Red=1, Blue=2, and Green=3. Nodes represent the countries and the relationship between them are represented by edges.

**Figure 5:**
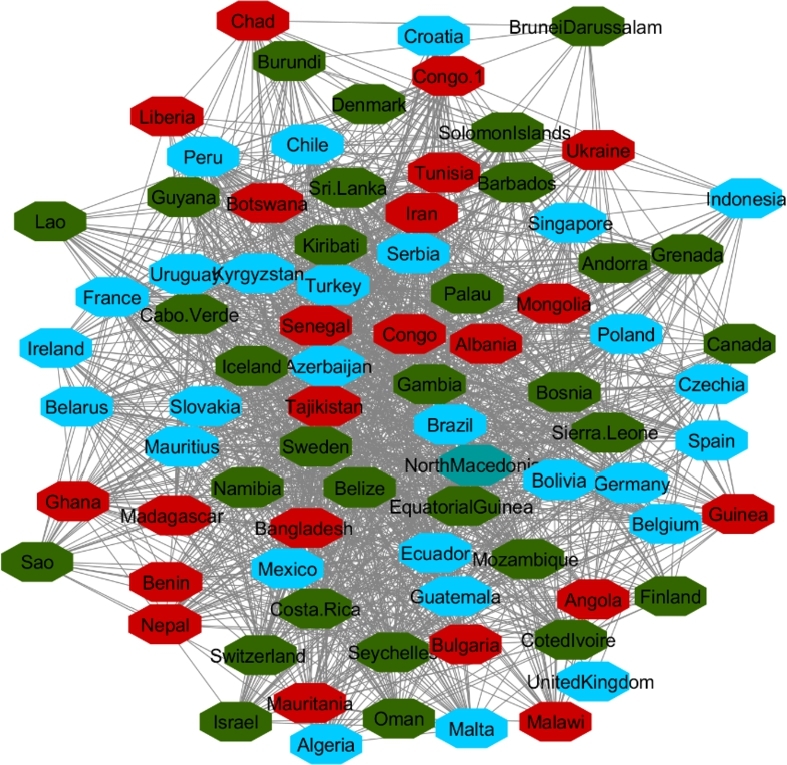
Voice and Accountability levels for Cluster 1 where Red=1, Blue=2, and Green=3. Nodes represent the countries and the relationship between them are represented by edges.

**Figure 6:**
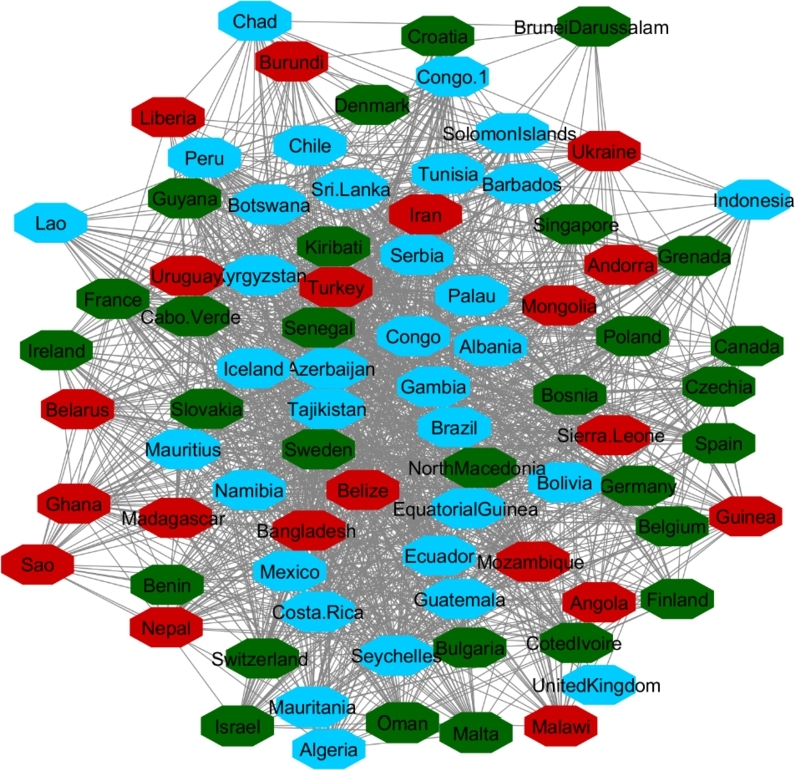
Inflation levels for Cluster 1 where Red=3, Blue=2, and Green=1. Nodes represent the countries and the relationship between them are represented by edges.

Fig. 4, Fig. 5 and Fig. 6 visualize the differences between stable and unstable countries in Cluster One. Since the data for each indicator contained range of numbers, we categorized the data points into different levels for better comparison/visualization. For this visualization, we categorized the Happiness Index, inflation, and VA to high, medium, and low levels. Level 1 (red) shows the*lowest rate* for each indicator and level 3 (green) shows the *highest rate* for the indicator. For example, since Level 1 represents the worst-case scenario, it shows the lowest rate of happiness and VA, and the highest rate of inflation whereas Level 3 shows the highest rate of happiness and VA and the lowest rate of inflation.

Manual inspection of Fig. 4, Fig. 5, and Fig. 6 shows that countries that are recognized as unstable have higher inflation and the lowest rate of happiness and the lowest rate of VA. On the other hand, stable countries stand for the low inflation rate, high level of happiness and a high level of VA.

**Figure 7:**
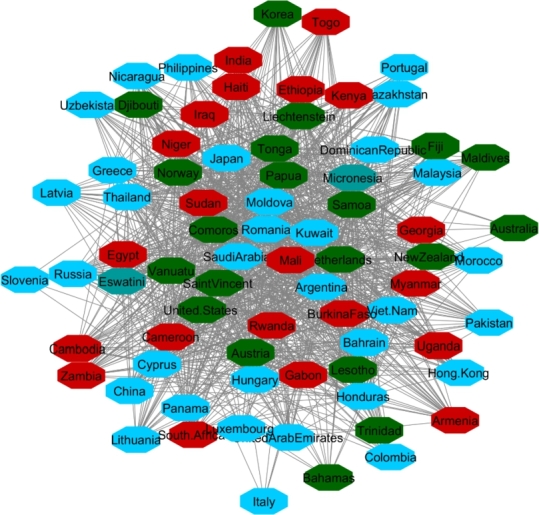
Happiness levels for Cluster 2 where Red=1, Blue=2, and Green=3. Nodes represent the countries and the relationship between them are represented by edges.

**Figure 8:**
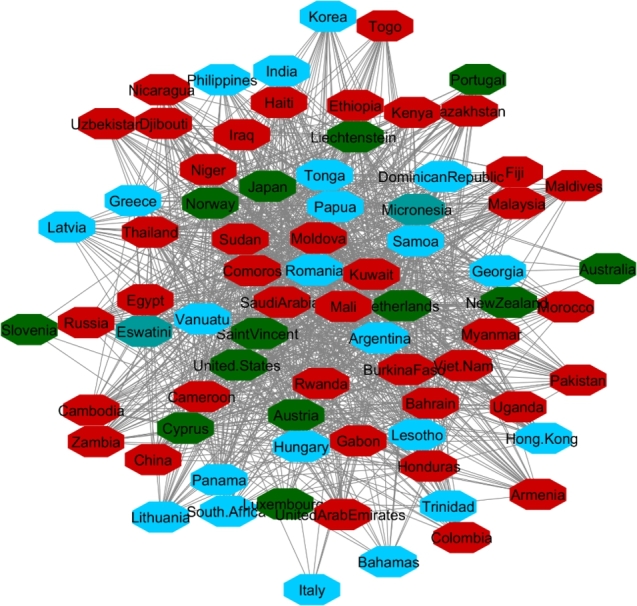
Voice and Accountability levels for Cluster 2 where Red=1, Blue=2, and Green=3. Nodes represent the countries and the relationship between them are represented by edges.

**Figure 9:**
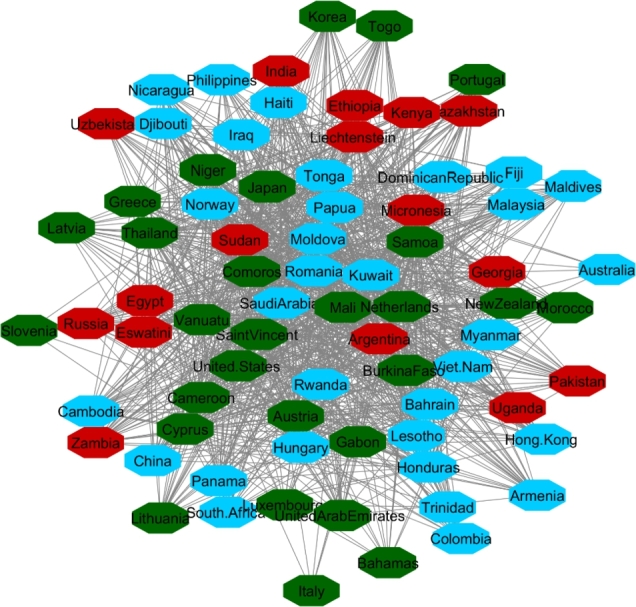
Inflation levels for Cluster 2 where Red=3, Blue=2, and Green=1. Nodes represent the countries and the relationship between them are represented by edges.

In Cluster Two, Norway, New Zealand, Austria, Japan, and Netherlands (considered as stable countries) and Hong Kong, Iraq, Myanmar, Egypt, Sudan (unstable countries) were in the same network communities. We applied the same procedure as Cluster One to Cluster Two and display the results in Table 2, Fig. 7, Fig. 8, and Fig. 9. The results show the same patterns as the results in Cluster One. Unstable countries (orange colors) mostly have high inflation, low level of happiness, and low level of VA compared to stable countries (green colors). Eswatini (Swaziland) and Micronesia did not have any data for the rest of indicators other than HDI.

**Table 2:**
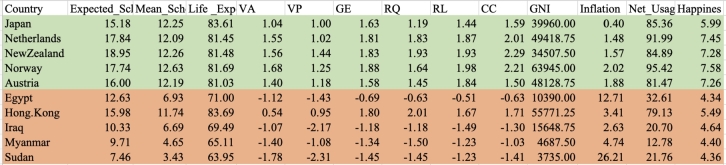
Cluster Two comparison between most stable and least stable countries.

**Figure 10:**
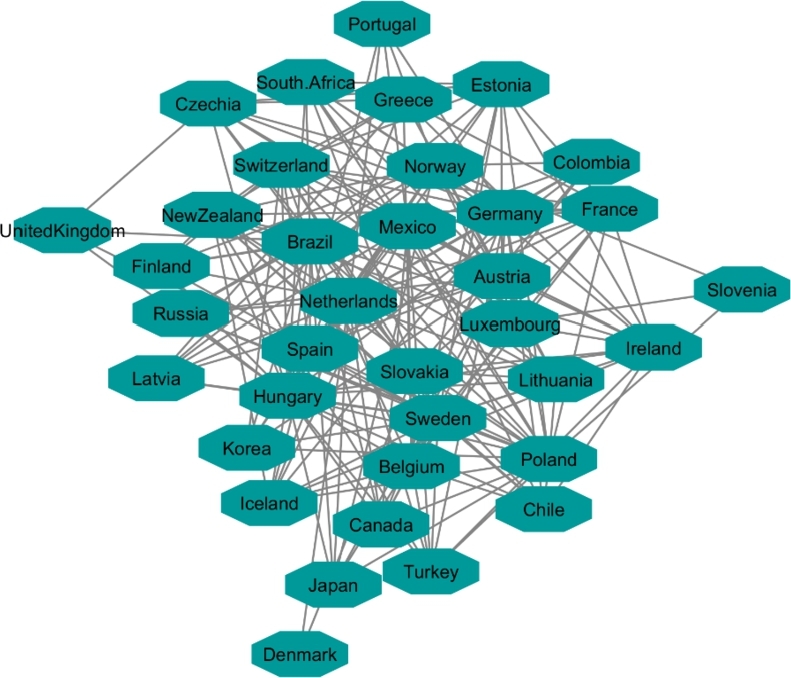
Network map of the OECD member states based on their HDI values.

**Figure 11:**
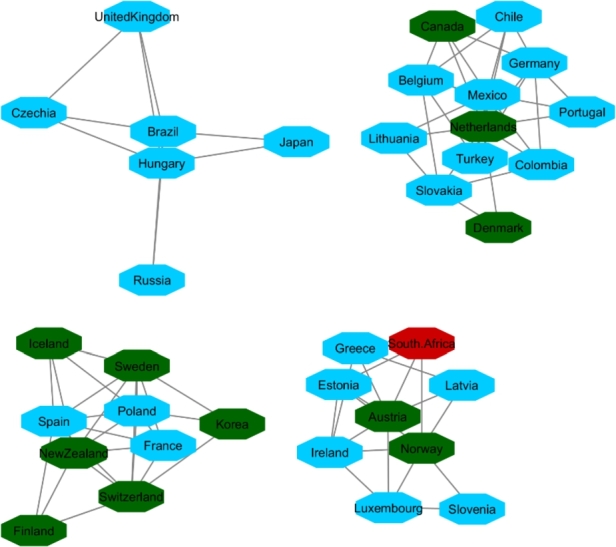
Happiness levels for OECD where Red=1, Blue=2, and Green=3. Nodes represent the countries and the relationship between them are represented by edges.

**Figure 12:**
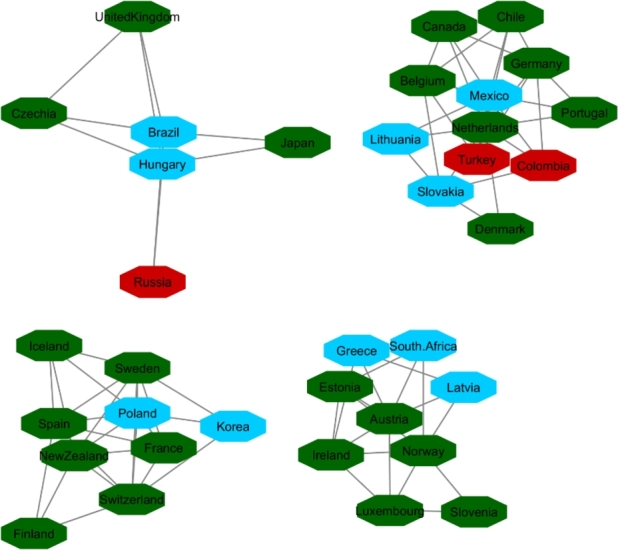
Voice and Accountability levels for OCED where Red=1, Blue=2, and Green=3. Nodes represent the countries and the relationship between them are represented by edges.

**Figure 13:**
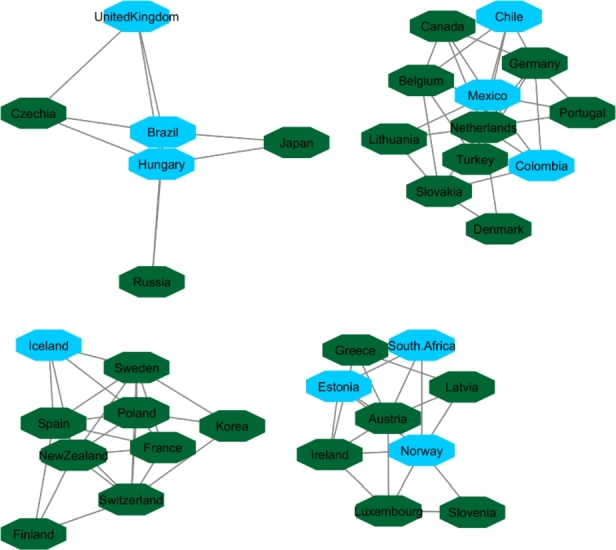
Inflation levels for OECD where Red=3, Blue=2, and Green=1. Nodes represent the countries and the relationship between them are represented by edges.

**OECD Network**:

Initially, there were 40 countries in OECD resource and after applying a 90% correlation coefficient of similarity in countries' HDI, 36 countries constructed a network (Fig. 10). Inputting all indicators in communities extracted from the OECD network showed that the changes in indicators during years for these countries followed the same patterns. For this network, we followed the same order as HDI's network for categorizing the indicators. Level one (red) shows the worst rate, and level three (green) shows the best rate for corresponded indicators. Since we compared inflation, VA and happiness, level one indicates high rate of inflation, low rate of VA, and happiness. Level three indicates low rate of inflation, high rate of VA and happiness. Fig. 11, Fig. 12, and Fig. 13 showed OECD's countries are in level two or three in the sense of happiness (except South Africa), VA (except Turkey, Russia and Colombia) and inflation. It is necessary to mention that Turkey, Mexico, France, Chile, and Brazil are members of the OECD countries and also had protests within the analyzed timespan or shortly thereafter. In the next section (Protest Network), we also included these countries in the list of protesters countries for further analysis.

**Protester Network**:

By looking at countries that within the analyzed timespan or shortly thereafter have conflict or protests in their society, we targeted 22 countries. Countries are in different regions with different political stands. Turkey, Mexico, Brazil, Chile, and France are OECD member states. Afghanistan, Iran, Iraq, Congo, Cuba, Egypt, Hong Kong, Lebanon, Libya, Myanmar, Sudan, Tunisia, Ukraine, Venezuela, Chad, Syria, and Yemen represent a variety of regions and political systems. The reasons behind the protests varied in the sense of political, economic, or social issues.^18^^,^^19^ We do not analyze pandemic-era protests.

**Figure 14:**
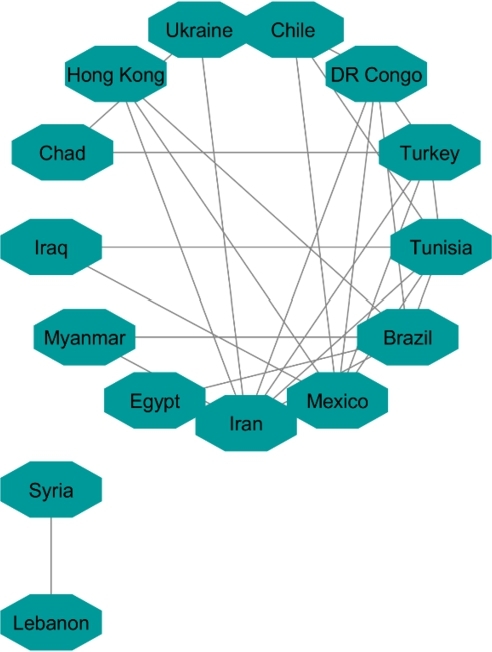
Protesters Network based on their HDI values.

**Figure 15:**
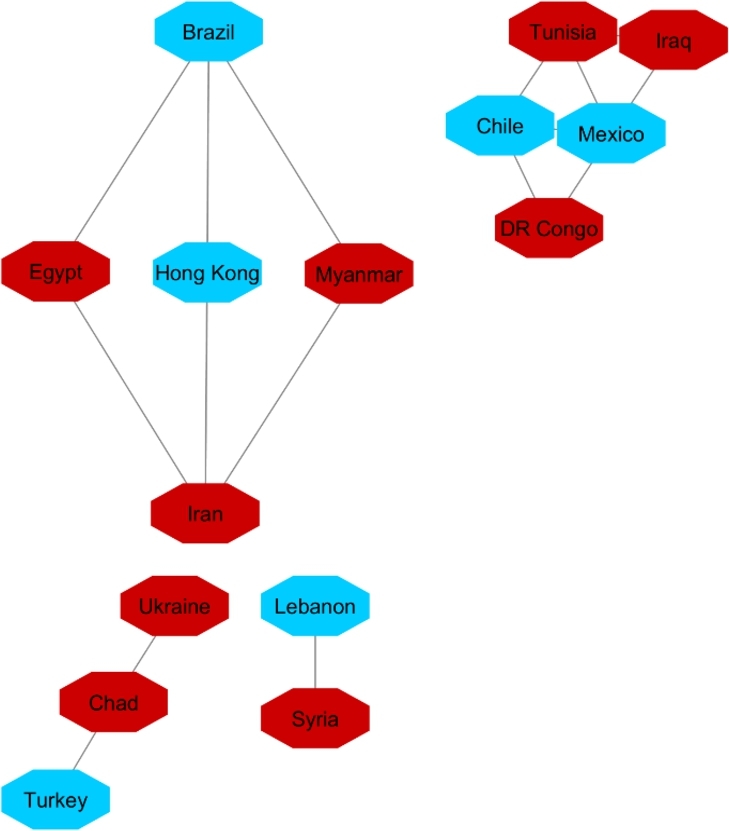
Happiness levels for Protest countries where Red=1, Blue=2, and Green=3. Nodes represent the countries and the relationship between them are represented by edges.

**Figure 16:**
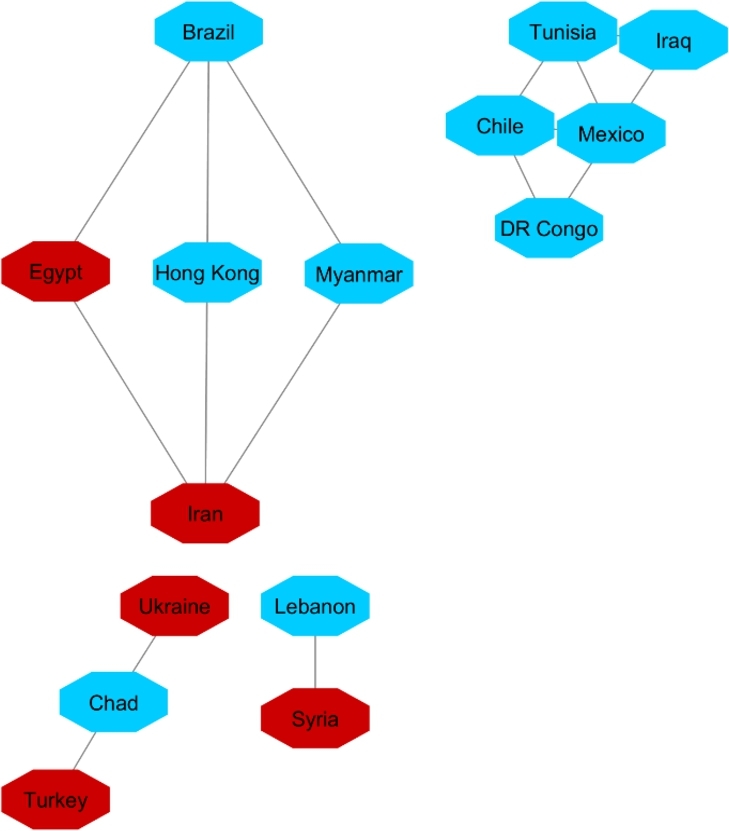
Inflation levels for Protest countries where Red=3, Blue=2, and Green=1. Nodes represent the countries and the relationship between them are represented by edges.

**Figure 17:**
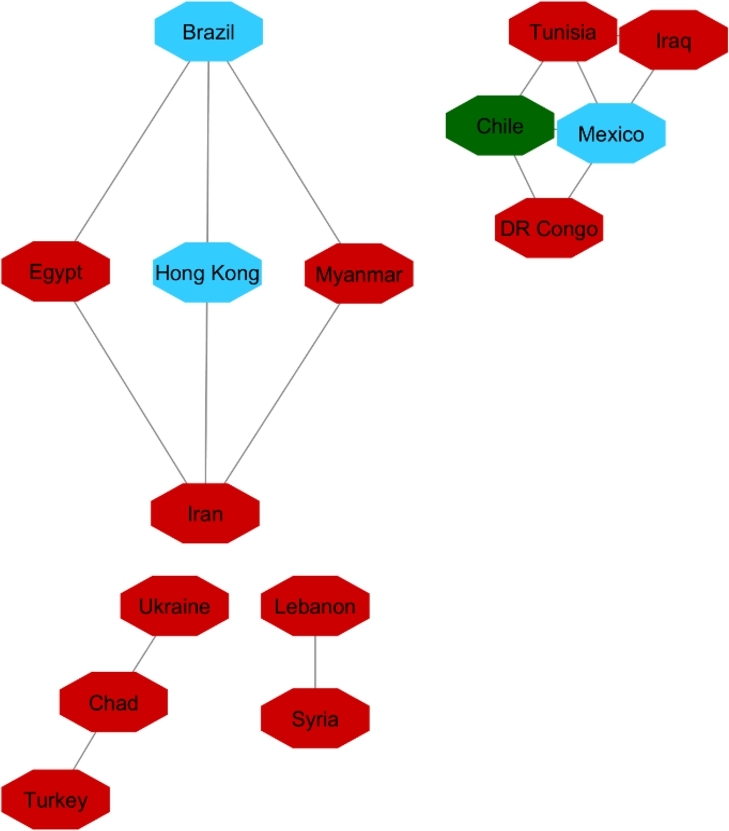
Voice and Accountability levels for Protest countries where Red=1, Blue=2, and Green=3. Nodes represent the countries and the relationship between them are represented by edges.

The same as with the two other analyses, we apply 90% as the coefficient of correlation for these countries' HDI's similarity. Fifteen countries constructed the network (Fig. 14). For cluster enrichment analysis, we categorized happiness, inflation and VA into three levels. The same as two other networks, level one (red) showed the highest rate of inflation, the lowest rate of happiness, and the lowest rate of VA. Level two (blue) showed these indicators at a moderate rate and level three showed that inflation as the lowest, and happiness and VA as the highest rate (green) (Fig. 15, Fig. 16, Fig. 17).

Fig. 15 showed that from countries involved in the protester network, none of them had the high level of happiness (level three, green). Regarding the happiness comparison, countries belong to OECD (Turkey, Mexico, Brazil, Chile) had level two of happiness (blue). Following in Fig. 16, Iran, Ukraine, Egypt, Syria and Turkey had high inflation rate and the rest of countries had level two of inflation rate. Coming to compare the VA indicator Fig. 17, 73% (11/15) of countries has the lowest rate of VA (red). From OECD list, Turkey was the country that had the lowest rate of VA and Chile has the highest rate of happiness among other OECD' countries.

**Network Analysis Implications**:

Social unrest has the potential and power to fundamentally alter a community and create institutional change but is not a requirement therefore (Kimmel and Kimmel, 1990). The selected indicators in this study are literature-informed indicators that might cause large-scale public protest in a country. For example, inflation is a sign of economic problems, low VA is a sign of a political or institutional issue, and the life ladder is an indicator describing life satisfaction.

The analysis shows that HDI is not a suitable indicator for assessing countries' well-being as a model for lived quality of life (RQ1). Our analysis shows that when using HDI to analyze patterns across countries, stable and unstable countries are grouped in the same cluster despite the high similarity required (90%) by the analysis to form the clusters. Considering actual similarities between countries, cluster enrichment shows that stable countries have the higher rate of education, health, GNI, high level of happiness, high level of VA, PV, GE, RQ, RL, CC, and low inflation rate. Unstable countries mostly stand in the opposite direction of stable countries. Several countries such as Chile, Turkey, Mexico, France and Brazil occur in both the analysis of OECD countries as well as the protesters network analysis. The rate of internet usage for all countries increases over time, however the difference between them is the rate of its growth. For example, the growth rate in internet usage between 2010-2017 in South Korea is 87%, and in Iraq, 20%.

Detailed analysis of the protester network shows that happiness and governance indicators such as VA play a major role in social crises compared to economic indicators, as well as inflation rates over time (RQ2). For example, Tunisia's activities showed that this country reports low levels of happiness and VA but only moderately high rate of inflation. In the following section, we analyze and report protester countries reflection of the J-Curve.

**Visualizing the J-Curve**:

Based on the same subset of 22 countries with recent protests as above, we attempt to model the Davies J-Curve. Fifteen countries constructed a network of four small clusters. We test for the J-Curve of these countries based on three indicators established as predictive in the previous analysis: rate of change in percent in inflation, VA, and happiness (x axis) over time (y axis). We use these three indicators as proxies of changes in political, economic and social satisfaction over time. In the case of modeling the Davies J-Curve based on these three indicators, we analyzed the three data curves against one another over the course of eight years. A country with increasing quality of life standards should see steady increases in VA and happiness, and decreases in inflation.

**Figure 18:**
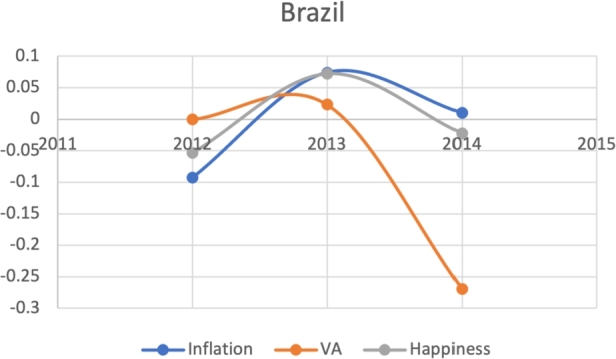
Rate of inflation, Voice and Accountability, and Happiness over time in Brazil: protest instance across 2015-2016.

**Figure 19:**
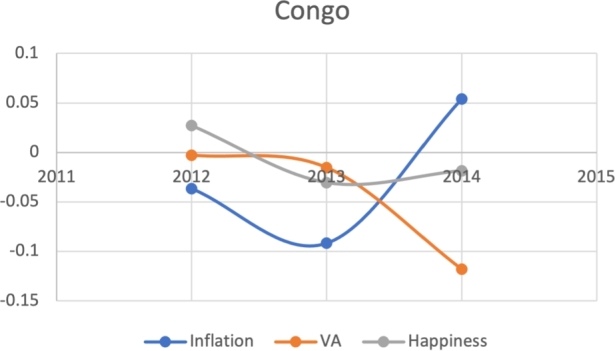
Rate of inflation, Voice and Accountability, and Happiness over time in the DR Congo: protest instance across: 2015.

**Figure 20:**
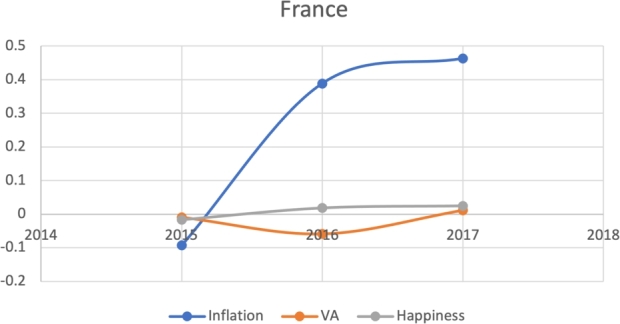
Rate of inflation, Voice and Accountability, and Happiness over time in France: protest instance across France: 2018.

**Figure 21:**
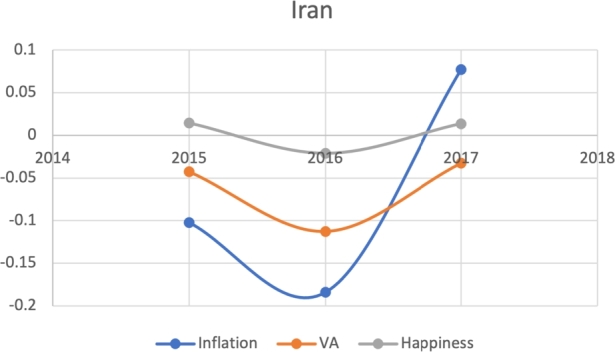
Rate of inflation, Voice and Accountability, and Happiness over time in Iran: protest instance across Iran: 2018.

**Figure 22:**
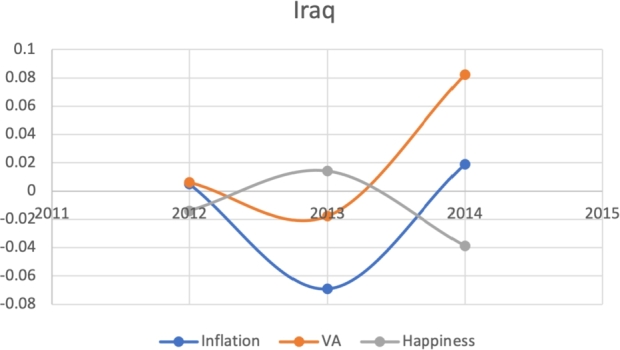
Rate of inflation, Voice and Accountability, and Happiness over time in Iraq: protest instance across Iraq: 2015.

**Figure 23:**
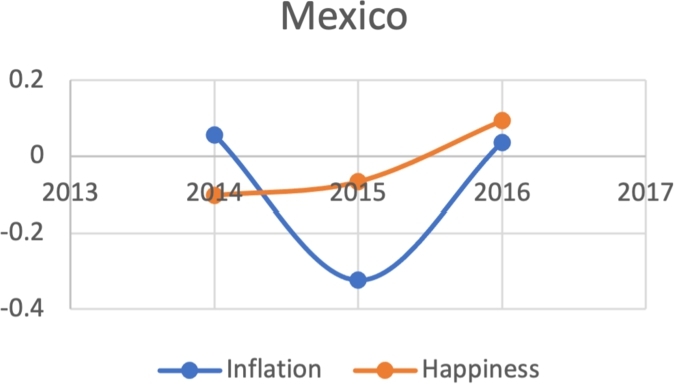
Rate of inflation and Happiness over time in Mexico.

**Figure 24:**
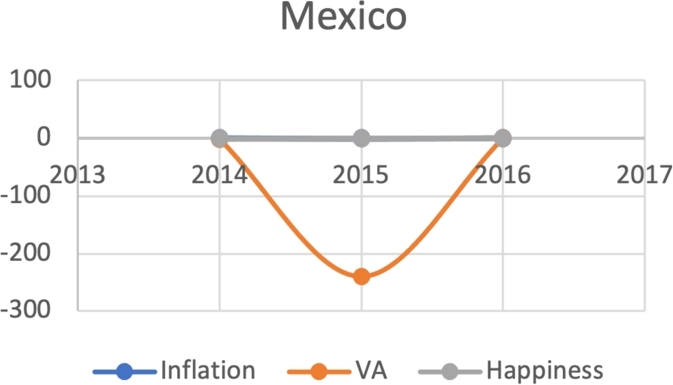
Voice and Accountability over time in Mexico.

**Figure 25:**
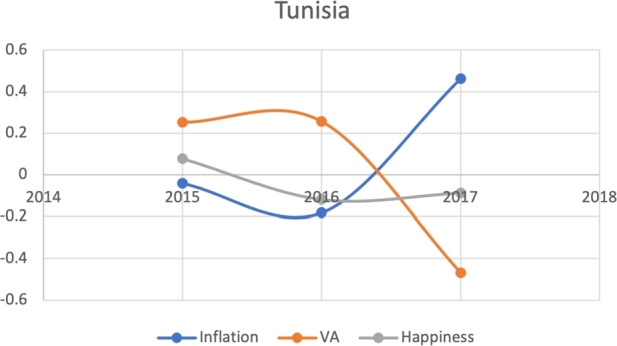
Rate of inflation, Voice and Accountability, and Happiness over time in Tunisia 2018.

**Figure 26:**
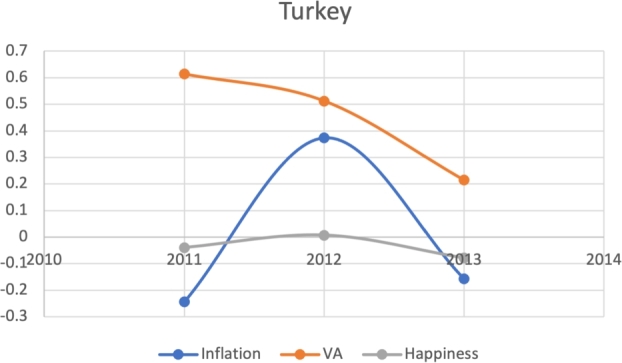
Rate of inflation, Voice and Accountability, and Happiness over time in Turkey 2013.

**Figure 27:**
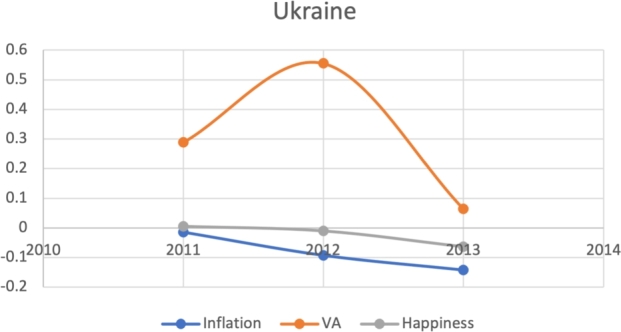
Rate of inflation, Voice and Accountability, and Happiness over time in Ukraine: protest instance across Iraq: 2014.

Due to the limitations in data availability regarding all indicators in this study (the data collected from 2010-2017), we tested the J-Curve for three years before the date of countries' protest. For example, if the protest happened in 2018, we checked the J-Curve for three years preceding 2018 which is 2015-2017. This leaves the ability, in other words, to test the J-Curve for the countries that had protests between 2014-2018. Based on the beforementioned data limitations, we could not assess for the J-Curve for Hong Kong (2019), Chad (2019), Syria (2011), Lebanon (2019), Myanmar (2007), Egypt (2019) and Chile (2019). The J-Curve graphs for Brazil (Fig. 18), DR Congo (Fig. 19), France (Fig. 20), Iran (Fig. 21), Iraq (Figure 22, Figure 26, Figure 27), Mexico (Figure 23, Figure 24), and Tunisia (Fig. 25), Turkey (Fig. 26), and Ukraine (Fig. 27) are shown below:

With reviewing the data from these countries, we could see that most countries experienced a precipitous drop(raise) over time on at least one indicator that is contrary to maintaining an expected quality of life (RQ3/4). All countries saw a rate change in happiness that was negative in the three years in advance of their most notable protest. Tunisia and Congo saw drops in VA and raises in inflation over time. In Mexico (Figure 23, Figure 24) the change over time in VA was so drastic (a 200% drop) that it defies comparison across indicators; inflation increased during the same time period.^20^ In Brazil, Turkey, and Ukraine, VA dropped most noticeably whereas in Iran, France, Iraq, and Mexico increases in inflation appear to be the major driver.

## 5 Discussion/conclusion

During periods of economic and social growth, the general population has a certain expectation that there is going to be a brighter future ahead of them. However, social unrest demanding institutional change, economic stability and demographic respect are a feature of the early 21st century. In his seminal article, James Davies argues that absolute changes in quality of life are less predictive of mass unrest than a perception that life should have been better. However, this theory could not have anticipated the advent of the consumer internet. In the digital era, an open question remains on how people are integrating comparison of living standards into their lives. When the world can be accessed at the touch of a button, against whom is a quality of life benchmark made?

This work posits that large, abstracted measures of quality of life like the Human Development Index are insufficient as an indicator for understanding the likely direction of a society. While these indicators can describe the expected and average life in a country over time, they cannot assess what it is like living in the country over time (Ghislandi et al., 2019). We find that access to the internet is also not descriptor of how well individuals live but rather the rate of growth. This is unexpected as one could expect that access, and the digital social platforms that come with access, would encourage comparison and thus displeasure. The results of the cluster enrichment analysis suggest that countries whose constituents live well are countries with high institutional stability, low inflation, and high reported subjective well-being (RQ1/2).

The countries in which a recent protest occurred generally followed the J-Curve model (RQ3) but they did so on the basis of differing or multiple indicators (RQ4). This suggests that the J-Curve can be empirically assessed and put to use by researchers and practitioners in their analyses of political change and unrest. However, the data which must be assessed is varied, which makes the evaluation complex to use as a predictive model. Future work should investigate creating more sensitive composite indicators if the model is to be used predictively, or consider data that has more frequent intervals than yearly intervals to increase sensitivity of the analysis.

### 5.1 Limitations

With the advancement of technology, access to digital technology may be significant to include as a measurement of human development and well-being. Some models pursue sentiment-based indicators sourced from social media or new reports to try to plug the gap between survey data and lived experience (Lindner et al., 2015; Voukelatou et al., 2022). Social media analytics added to standard macro indicators is a good but lofty goal; whereas its integration would support assessment of countries missing data (e.g., those with open war like Syria), the amount of data compared to the possible benefit is a hard trade-off. Social media data also comes with its own biases - due to the nature of social media data collection, it is currently not possible to validate “ground truth” in a way that standard social research approaches can.

A final set of limitations comes from the data itself. We aware of issues of sample size. The countries in which it is most critical to assess life on the ground rarely have complete data; moreover, there are few standards for use in terms of indicators expressing how individuals are using the internet, or a unified gender quality indicator. Additional high quality data can only increase our understanding of the roots of unrest in society, however necessary metrics are still broadly lacking.

## Declarations

### Author contribution statement

Zahra Hatami: Conceived and designed the experiments; Performed the experiments; Analyzed and interpreted the data; Contributed reagents, materials, analysis tools or data; Wrote the paper.

Sue Yi: Contributed reagents, materials, analysis tools or data; Wrote the paper.

Margeret Hall: Conceived and designed the experiments; Analyzed and interpreted the data; Contributed reagents, materials, analysis tools or data; Wrote the paper.

### Funding statement

There was no funding support for this work.

### Data availability statement

Data will be made available on request.

### Declaration of interest's statement

The authors declare no conflict of interest.

### Additional information

No additional information is available for this paper.

## Footnotes

1 https://www.reuters.com/article/us-bolivia-politics/bolivians-take-to-the-streets-to-protest-political-arrests-idUSKBN2B82GU.

2 https://www.citylab.com/equity/2019/10/chile-protest-santiago-metro-public-transit-fare-inequality/600874/.

3 https://www.aljazeera.com/news/2017/9/12/protests-across-france-against-macrons-labour-reforms.

4 https://www.nytimes.com/2019/11/16/world/middleeast/iran-gas-price.html.

5 https://www.hrw.org/blog-feed/hong-kong-protests.

6 https://www.brandeis.edu/now/2019/december/iraq-protests-2019.html.

7 https://www.imf.org/external/pubs/ft/fandd/2021/08/economics-of-social-unrest-imf-barrett-chen.htm.

8 This work uses the terms cluster and community to describe cluster analysis output interchangeably.

9 https://ourworldindata.org/.

10 https://blogs.worldbank.org/opendata/what-does-life-expectancy-birth-really-mean.

11 http://hdr.undp.org/en/content/mean-years-schooling-males-aged-25-years-and-above-years.

12 https://www.insee.fr/en/metadonnees/definition/c1156.

13 https://www.gfmag.com/global-data/glossary/gdp-gni-definitions.

14 https://data.worldbank.org/indicator/NY.GDP.PCAP.PP.CD.

15 https://data.worldbank.org/indicator/NY.GDP.PCAP.PP.CD.

16 https://www.trackinghappiness.com/happiness-index-2018/.

17 https://www.mcc.gov/who-we-select/indicator/control-of-corruption-indicator.

18 https://www.economist.com/international/2019/11/04/why-are-so-many-countries-witnessing-mass-protests.

19 https://www.abc.net.au/news/2019-10-29/protests-around-the-world-explained/11645682.

20 2015 saw a national and international outcry based on the disappearance of 43 protesting students widely blamed on collusion between the local government and cartels. https://www.bbc.com/news/world-latin-america-29538079.
